# Clinical and economic outcomes of hospital pharmaceutical care: a systematic review and meta-analysis

**DOI:** 10.1186/s12913-020-05346-8

**Published:** 2020-06-01

**Authors:** Guohua Lin, Rong Huang, Jing Zhang, Gaojie Li, Lei Chen, Xiaoyu Xi

**Affiliations:** 1 China Pharmaceutical University, Nanjing, China; 2 The Research Center of National Drug Policy & Ecosystem, China Pharmaceutical University, No.639 longmian Avenue, Jiangning District, Nanjing, 211198 China

**Keywords:** Pharmaceutical care, Systematic review, Meta-analysis

## Abstract

**Background:**

Hospital clinical pharmacists have been working in many countries for many years and clinical pharmaceutical care have a positive effect on the recovery of patients. In order to evaluate the clinical effectiveness and economic outcomes of clinical pharmaceutical care, relevant clinical trial studies were reviewed and analysed.

**Methods:**

Two researchers searched literatures published from January 1992 to October 2019, and screened them by keywords like pharmaceutical care, pharmaceutical services, pharmacist interventions, outcomes, effects, impact, etc. Then, duplicate literatures were removed and the titles, abstracts and texts were read to screen literatures according to inclusion and exclusion criteria. Key data in the literature were extracted, and Meta-analysis was conducted using the literature with common outcome indicators.

**Results:**

A total of 3299 articles were retrieved, and 42 studies were finally included. Twelve of them were used for meta-analysis. Among the 42 studies included, the main results of pharmaceutical care showed positive effects, 36 experimental groups were significantly better than the control group, and the remaining 6 studies showed mixed or no effects. Meta-analysis showed that clinical pharmacists had significant effects on reducing systolic blood pressure and diastolic blood pressure and shortening hospitalization days (*P* < 0.05), but no statistical significance in reducing medical costs (*P* > 0.05).

**Conclusion:**

Clinical pharmacists’ pharmaceutical care has a significant positive effect on patients’ clinical effects, but has no significant economic effect.

## Background

Pharmaceutical care is the direct, responsible provision of medication-related care for the purpose of achieving definite outcomes [1]. Though identifying, solving and preventing medication problems, finding out prescription errors and medication-related injuries by clinical pharmacists, incidence of adverse events and rehospitalization rates could be reduced. Patient adherence of the treatment could be significantly improved and possible harm due to medication problems had been reduced after patients received their medication instructions [2]. Medication education and treatment advice from clinical pharmacists could also shorten hospital stay [3].

Studies have shown that hospital pharmaceutical care had great value in clinical and economic aspects. In a diabetes management team, participation of clinical pharmacists led to the reduction of hemoglobin, cholesterol and blood pressure in patients as well as the significantly lower cost of medication for each patient [4]. A study showed the implementation of antifungal practice guidelines by a clinical pharmacist, member of an ICU team, resulted in a 50% cost reduction in expenditure on antifungal agents [5]. However, whether there was a direct connection between this service and the improvement of patient health had been discussed. Meanwhile, costs of running pharmacy service and its economic benefits were at issue in some countries. These worries impeded the development of hospital clinical pharmacy and its universal implement. Among factors mentioned above, lack of strong, direct evidence is one potential barrier.

Although many studies noticed the clinical and economic outcomes of hospital pharmaceutical care, few systematically demonstrated and validated the effectiveness of hospital pharmaceutical care. Due to flaws in experimental design and source of literature, non-randomized controlled trials, low methodological quality of included studies or unconvincing experimental data, evidence on effectiveness and validity are still insufficient. Therefore, it is necessary to explore its clinical and economic outcomes from the scope of a more general perspective. In the present study, a systematic review and meta-analysis for pooling statistical power was conducted to systematically evaluate the clinical and economic outcomes of hospital pharmaceutical care.

## Methods

### Search strategy

Two researchers searched for relevant articles published in databases including Pubmed by Medline, Embase, Cochrane and CINAH (January 1992 to October 2019). Key words included pharmaceutical service/care/intervention, pharmacy service/care/intervention, pharmacist service/care/intervention and clinical outcomes, evaluations, effects, assessment, outcomes, practice. And it is supplemented by such truncated words as “service *”, “analysis *”, “evaluate *”, “effect *”, “Pharmac *”, “intervene *”, “practi *”, “impact *”. The retained researches were supplemented by access to monographs, reviews, references to published articles, and recently published Chinese and English journal articles. Two reviewers independently searched and discussed and resolved discrepancies.

### Inclusion and exclusion criteria

Studies would be included when interventions or participation of clinical pharmacists were considered with detailed descriptions of services they provided. The research setting should be conducted in hospitals. The research conducted should involve intervention groups and control groups who received routine care or non-interventions from clinical pharmacists. The clinical outcomes or economic outcomes of the interventions should be evaluated. Studies only abstracts available were excluded.

### Data extraction and validity assessment

The data extraction was independently carried out by the researchers using a standard electronic form Microsoft Excel 2016 and the extracted data was checked by two researchers. According to the Cochrane systematic review guidelines, combined with the aim of this study and quality assessment requirements, extracted data in the feature tables included:

1. For numbered lists Literature characteristics (Table 1): author, publication year, country, sample source, interventions, primary outcomes and effects.
2. Methodological quality assessment table: correct randomization method, hidden allocation scheme, blindness method, whether there is bias due to missing data.

**Table 1:** Literature Characteristics

NO.	Author (year) country	Sample description	Pharmacist interventions	Primary outcomes	Effect
1	Bill et al [6] (1992) US	From 1984 to 1987, patients admitted to the hospitals, 432 admissions on four general medicine services over 12 months	Provide a brief guidebook, a booklet on cost strategies and common expenses, detailed temporary bills, and information on the number of days hospitalized and the usual hospitalization costs	Hospitalization days, diagnosis-related group adjustment costs, direct standardization charges	Positive
2	Maryanne [7] (1992) US	Not reported	Change dosage and content of prescriptions; discontinue use of prescriptions	avoided costs	Positive
3	Carter et al [8] (1997) US	Adult patients with primary hypertension in any ethnic group in a Christian medical clinic, 25 in the intervention group and 26 in the control group	Visit patients every 3–5 weeks and get drug supplement; measure blood pressure and pulse; inquire about adverse drug reactions and improve adherence to treatment at each follow-up; write a complete progress record; evaluate the patient’s current medical treatment and understanding of lifestyle changes; record all patient data and send a copy to the patient’s physicians for review; pharmacists and physicians involved at that time contacted and provide services for patients who needed to change medications; standardize patient education, distribute brochures, visual materials, and verbal instructions	Blood pressure, visits, medication costs	Positive
4	GL et al [9] (1997) US	Patients who received parenteral antibiotics from January to March 1994 at the Portland Hospital in the US, 141 in the intervention group and 111 in the control group	Provide patient-specific, antibiotic-related advice to the attending physician (by a team of infectious disease researchers and clinical pharmacists)	Reuse of antibiotics, mortality, per capita antibiotic costs	No effect
5	Gums et al [10] (1999) US	In the adult patient with uncontrolled dyslipidemia defined by the 2009 Canadian Dyslipidemia Guidelines, 43 were in the intervention group and 44 in the control group.	Determine the best intravenous antibiotics; advise on antibiotic treatment and monitoring	Hospitalization time, average hospitalization days, hospitalization costs, patient mortality	Positive
6	Dager et al [11] (2000) US	A 400-bed teaching hospital, patients older than 18 years old, who received warfarin for the first time, the 60 patients hospitalized in 1992 were the control group, and 60 patients hospitalized in 1995 were intervention groups.	Review the patient’s medication history; provide written consultations daily on the medical charts of patients with warfarin dosing recommendations	Hospitalization days, average INR at the time of discharge from the INR	Positive
7	Canales et al [12] (2001) US	From May to December 1997, any psychiatric patient admitted to the Austin National Hospital with acute psychotic symptoms, 45 in the intervention group and 48 in the control group	Participate in treatment group meetings; perform baseline assessments and weekly observations; provide medication recommendations; obtain medication history; review drug administration daily records; monitor adverse drug reactions; conduct medication education classes; consult patients before they leave the hospital	Average medical treatment cost per patient during hospitalization	Positive
8	Brook et al [13] (2003) Netherlands	From April 2000 to April 2001, patients who went to pharmacies to purchase antidepressant drugs, 64 in the intervention group and 71 in the control group	Introduce drugs and drug efficacy to patients and discuss ways of drug use; provide videos related to pharmaceutical education and patient counsels	The number of positive drug attitudes	Positive
9	Bolas et al [14] (2004) UK	The hospitalized patients in the Antrim district hospital were 81 in the intervention group and 81 in the control group	Prepare accurate drug records after full review of current drug use; drug counseling; provide medication record forms to inform patients on how to take medication; provide medications detailing changes in drug treatment; release letter (general practitioner faxed to patient on the day of discharge community pharmacist); provide helpline for medicines	Average mismatch rate between discharge prescription and household medication, average error rate of drug treatment knowledge	Positive
10	Carter et al [15] (2009) US	Men and women aged over 21 in 6 clinics diagnosed as essential hypertension, taking 0 to 3 compression medicines, 192 intervention groups and 210 control groups	make drug therapy recommendations to physicians based on national guidelines	Blood pressure, blood pressure control rate	Positive
11	Wong et al [16] (2010) Singapore	From 2006 to 2007, patients in the general medical and surgical departments of a 1200-bed nursing teaching hospital in Singapore who initially started taking warfarin for deep vein thrombosis, pulmonary embolism or atrial fibrillation, intervention group 144 and control group 26	Support the commencement and titration of warfarin anticoagulant services; provide written counseling and discuss the case with the doctor; recommend daily warfarin’s dose; check and monitor patients’ International Normalization Ratio until they are ready for discharge; recommend discharge doses and appointment dates for anticoagulation clinics	Hospitalization days, international standardized ratio, average number of days discharged	Positive
12	Hammad et al [17] (2011) Jordan	From March to November 2009, patient enrolled in the family medical clinic of Jordan University Hospital, 110 in the intervention group and 89 in the control group	Provide a 30-min consultation before meeting with a physician	Triglycerides, high-density lipoprotein cholesterol, blood sugar, Blood pressure	Positive
13	Shen et al [18] (2011) China	Between July 2009 and April 2010, inpatients in two separate respiratory wards at three teaching hospitals, 178 in the control group and 176 in the intervention group	communicate with physicians; make recommendations on treatment options	Hospitalization costs, antibiotic costs, hospitalization days	Positive
14	Mousavi et al [19] (2012) Iran	Patients who had at least one significant risk factor or at least two related risk factors in the kidney ward of the Iranian Khomeini Hospital Complex for 6 months, 375 in the intervention group and 236 in the control group	escort physicians in the ward and gives suggestions	Appropriate and inappropriate stress ulcer prophylaxis management per patient cost	Positive
15	Shah et al [20] (2012) US	Between 2010 and 2011, diabetes patients over 18 years old in a public hospital and health care system, 31 in the intervention group and 21 in the control group	Consult on routine care and post-discharge diabetes drug dosage, side effects and clinical benefits; concurrent diabetes mellitus symptoms, hypoglycemia, healthy eating, exercise and reduced use guidelines emotional education; follow-up after discharge	Cholesterol, lipoprotein, Blood pressure, glycated hemoglobin	Positive
16	Zhang et al [3] (2012) China	The pediatric patients with neurological diseases, respiratory diseases or digestive diseases in the Second Hospital of HuaXi, Chengdu, China, 76 in the intervention group and 74 in the control group	Answer questions from doctors and nurses; provide treatment advice; prevent medication errors	Hospitalization days, medical cost per patient, readmission rate	Mixed
17	Cies, Varlotta [21] (2013) US	From January 2007 to August 2008 in St. Christopher’s Children’s Hospital, 29 in the intervention group and 22 in the control group	Specialized clinical pharmacists monitor and adjust drug dosing; monitor initial and subsequent tobramycin levels	Total cost, hospitalization cost, dose adjustment cost, average hospitalization days	Positive
18	Ho et al [22] (2013) UK	From January 1, 2004 to March 31, 2007, patients admitted to the Royal Hospital of Colombia had 333 interventions and 1228 patients in the control group	The presence or absence of one or more clinical pharmacy notes recorded in the inpatient record during the ICU admission	Complete cohort mortality	Positive
19	Chilipko, Norwood [23] (2014) US	From January 1, 2009 to January 1, 2011 in a community teaching hospital, patients are over 18 years old and receive warfarin for at least 3 days, 125 in the intervention group and 108 in the control group	Provide anticoagulation management services for warfarin; daily monitor warfarin dosage	In-hospital average treatment time, hospitalization period average number of days for achieving INR goals, total incidence of bleeding, average albumin	Mixed
20	Grimes et al [24] (2014) UK	Between July 2010 and May 2011 Adult patients at the Tallaght Hospital in Dublin, Ireland, 112 in the intervention group and 121 in the control group	Medication reconciliation and prescription exams; understand the inpatient history of medication	Errors in medication, changes in cumulative drug adaptability index before admission to hospital and after discharge	Positive
21	Joost et al [25] (2014) Germany	From August 2008 to July 2010 at the Erlangen University Hospital, patients who were able to visit repeatedly for outpatients with kidney disease and hypertension, 35 in the intervention group and 39 in the control group	Provide additional inpatient and outpatient pharmaceutical care; counsel by a specialized clinical pharmacist	Percentage of days of correct dosage, pc adhesion rate	Positive
22	Tan et al [26] (2014) Australia	From December 2011 to January 2013, in two general practice clinics in Melbourne, Australia, 62 patients with one or more risk factors for medication-related problems, sample size 62 people	Provide face-to-face consultations; interview in private clinics for about 30–60 min; resolve issues related to identifying drugs at home	Patient’s rate of adherence to their medication, health score	Positive
23	Vervacke, Lorent, Motte [27] (2014) Belgian	From September 2009 to March 2012, in a Belgian urban academic hospital who aged 75 or older with a history of venous thromboembolism or cancer, 336 before the intervention, 431 after the intervention	Provide education for specific physicians and nurses; disseminate teaching tools to summarize guidelines and reminders for venous thromboembolism prevention	Number of patients at risk of venous thromboembolism	Positive
24	Xin et al [4] (2014) China	From January to December 2013, in Zhejiang Province Tongde Hospital who is less than 18 years old, diagnosed as type 2 diabetes, 420 before the intervention, 429 after the intervention	A full-time experienced pharmacist served in the team	Hemoglobin, lipoproteins, triglycerides, Blood pressure, hospitalization days, medication costs	Positive
25	Zhang et al [28] (2014) China	From 2011 to 2012, inpatients undergoing cleansing or decontamination operations in the Department of Urology, 174 before intervention and 196 after intervention	Monitor drug information and make medical records in real time through the hospital information system; establish standards for the administration of preventive antibiotic prescriptions through hospital management	Drug costs, antibiotic prevention delay days	Positive
26	Campo, Roberts, Cooter [29] (2015) Australia	University of South Australia’s Higher Education Hospital Flinders Medical Center admitted to the respiratory ward in July 2010, 31 patients in the intervention group and 30 in the control group	Measure 4 whole-day glycemic profiles 24 h per phase (Non-diabetic patients with chronic obstructive pulmonary disease); daily test 4 whole-day glycemic profiles (patients with chronic obstructive pulmonary disease); blood glucose levels are at 700 h, 1200 h, 1700 h (before meals) and 2100 h for routine monitoring	Achieve daily minimum blood glucose monitoring level, cross-time blood glucose level test	Positive
27	Delpeuch et al [30] (2015) French	Department of Hematology/Oncology, Affiliated Hospital of Medical School, Patient with solid tumor (excluding lung cancer), sample size 552	Comprehensive drug review (chemotherapy, supportive care and outpatient treatment)	Drug related issues	Mixed
28	Obarcanin et al [31] (2015) Yugoslavia and Germany	Two paediatric clinics in the Krefeld region of Germany and Sarajevo, Bosnia and Herzegovina, 39 in the intervention group and 26 in the control group	Provide access to pharmaceutical services monthly and record clinical data during visits; patients in the intervention group measured at least 4 times daily blood glucose; assess drug-related needs and identify problems; develop an individualized pharmaceutical care plan for each patient; pharmacists discuss the drug care plan with physicians	Glycated hemoglobin	Positive
29	Wolf et al [2] (2015) UK	From September 2012 to December 2013, 269 mental patients were sent to the psychiatric department, 131 in the intervention group and 134 in the control group	Provide detailed medication reconciliation at admission and medication reviews at discharge and 3 months after discharge; two clinical pharmacists follow each week during hospitalization	Change in drug fitness index, number of medication-related issues	Positive
30	Burnett et al [32] (2016) US	All patients who received heparin-induced thrombocytopenia per-intervention (10/1/2009–9/ 30/2010) and post-intervention (10/1/2010–9/30/2011) had 167 patients before the intervention, 104 people after the intervention	The pharmacy-driven 4 T score (4 T pretest probability score) intervention	Calculated 4 T score, number of patients with major bleeding, number of patients with thrombotic events, average cost per patient	Positive
31	Gallagher et al [33] (2016) UK	From June 2011 to June 2012, hospitalized patients in an 810-bed teaching hospital in Ireland, 361 people in the intervention groups and 376 people in the control group	Provide medication reconciliation, deployment of clinical decision support software; formulation of a pharmaceutical health plan	Total cost, adverse drug reactions	Mixed
32	Khalil V et al [34] (2016) Australia	Inpatients in general hospitals in a hospital in Australia, 56 in the intervention group and 54 in the control group	Pharmacist medication guidance	Medication errors, the severity of prescribing errors	Positive
33	Phatak et al [35] (2016) US	From November 2012 to June 2013, patients discharged from the 894-bed academic medical center or western memorial hospital of the Northwest Memorial Hospital in Chicago, Illinois, 137 in the intervention group and 141 in the control group	Face-to-face medication reconciliation; a patient-specific pharmaceutical care plan; discharge counseling; post discharge phones call on days 3, 14, and 30 to provide education and assess study endpoints	High-risk average, number of days admitted to hospital/emergency, drug-related readmissions, general hospital admissions	Positive
34	Watersl et al [36] (2017) US	A hospital in the US had been discharged from the emergency department and had been discharged, and was later found to be a positive bacterial pathogen in the blood culture. 138 were in the intervention group and 107 in the control group	Provide advice on proper antibiotic selection, dosage, route, and duration; evaluate the efficacy of excretion antibiotics and intervene when pathogen-antibiotic mismatches are found; reduce workload of physicians’ in emergency departments; improve antimicrobial management experience in the culture process	Proportion of patients receiving appropriate antibiotic treatment, rate of admission or readmission within 90 days, the number of 90-day cases of illness	Positive
35	Sloeserwij et al [37] (2019) Netherlands	From January 2013 to May 2015, 11,928 high-risk patients were included	10 specially trained non-dispensing pharmacists took integral responsibility for the pharmaceutical care. They provide a wide range of medication management services at the patient level (e.g. clinical drug review) and the level of practice (e.g. quality improvement projects).	the number of medication-related hospitalisations	Possitive
36	Schumacher et al [38] (2018) US	From November 2009 toAugust 2010, Clinical pharmacists visited and intervened 111 patients with chronic heart failure.	Clinical pharmacists improve hospitalization rates and 30-day readmission rates for heart failure through more frequent follow-up and improved access to care. Clinical Spaces have been established for clinical pharmacists including patient visit rooms and independent provider schedules. But the need for other chronic comorbidity quickly became apparent, requiring expanded services and the role of clinical pharmacists within months of the establishment of the practice.	readmission rates	Possitive
37	Korcegez et al [39] (2017) Northern Cyprus	From October 2013 to July 2015,152 patients were treated in the diabetes clinic of a public hospital in gazimagus, northern Cyprus. The patients were divided into two groups: intervention group (75 cases) and usual care group (77 cases).	Each patient scheduled a meeting with the study clinical pharmacist on the same day as the doctor’s appointment. The pharmacist interviewed the patient in an independent office next to the doctor’s office. The intervention group conducted 5 consecutive visits with a pharmacist every 3 months and reviewed the medication and treatment plan.	change in A1c	Possitive
38	Domingues et al [40] (2017) Spain	From April 2013 to November 2014, the study population included 42 patients receiving treatment from the third hospital pharmacy outpatient department and receiving antiretroviral therapy.	Drug treatment was followed up using the dader method. Interviews were conducted every 2 months. During each interview, the patient’s medication, health problems, and modifiable cardiovascular risk factors were assessed. Direct drug care interventions to patients when lifestyle changes or improved treatment compliance are required. If treatment needs to be evaluated, the doctor receives a written report.	changes in cardiovascular risk	Possitive
39	Ospina et al [41] (2017) Colombia	From November 2011to June 2014, 92 patients were randomly divided into intervention group (43) and control group (49).	The pharmacist calls every week until the end of the study. During these calls, the pharmacist did the following: (a) conducting clinical assessments, assessing changes in mood, behavior, regular eating and sleep patterns, language and thinking; (b) emphasizing the importance of patient education and the identification and management of prodromal symptoms; (c) to explain the correct use of bipolar drugs; (d) promoting treatment compliance; and (e) promoting healthy eating and lifestyle habits.	hospitalizations and emergency service consultations, unscheduled outpatient visits, clinical evaluation of depression and mania	Mixed
40	Javaid et al [42] (2019) Pakistan	From August 2016to June 2017, there were 52 and 83 patients in the control and intervention arm, respectively	Pharmacological interventions involve working with doctors to identify drug-related issues, drug interactions, dose, frequency changes, and treatment transitions, whereas non- pharmacological interventions involve diet, lifestyle, and behavioral counseling.	glycemic (HbA1c), lipid controls.	Possitive
41	Shao et al [43] (2017) China	After strict screening, 120 patients were randomly divided into two groups. And one hundred ninety-nine patients completed the study	Interviews included face-to-face interviews (once every other month) and telephone follow-up (every month) until the end of the study. During the interview, the pharmacist discussed about their medication compliance, self-monitoring of blood sugar control, and exercise; explained side effects and possible drug interactions; and reminded them to see the doctor next time.	FBG, HbA1c, TC, the target attainment rates of HbA1c, BP	Possitive
42	Juanes et al [44] (2018) US	From January 2012 and February 2013, patients were allocated in a 1:1 ratio of potential drug-related problems (intervention group) or administered as standard care (control group).	review the following aspects of the patient’s medication: (a) the indications of each drug are related to the patient’s condition; (b) the suitability, dose, plan, and treatment time of each drug are related to the patient’s age and / or clinical status (renal or liver function). In addition, therapeutic drug monitoring was carried out for drugs with narrow treatment range.. Follow up. Assess the effectiveness and safety of treatment based on standard clinical practice and objective patient data from clinical records.	drug-related negative outcomes	Possitive

When comparing the main outcomes of experimental groups and control groups, *p* < 0.05 was viewed statistically significant. When the primary outcomes of the experimental group were significantly better than the control group, it was marked as “positive”; and when there were no significant difference between the two groups, it was viewed as “no effect”. For studies evaluating multiple primary outcomes and not positive outcomes, those who presented at least one major positive outcome were considered as “mixed”.

### Meta-analysis

In this study, Stata 15 was used for meta-analysis. After calculating the number of studies with common outcomes, systolic blood pressure (SBP), diastolic blood pressure (DBP), medical cost, and hospitalization days remained for meta-analysis. The standard mean difference (SMD) was used as the effect quantity, the significance level (or) of the combined effect quantity test was 0.05, the significance level of the heterogeneity test was 0.1, and the overall estimate was expressed by the point estimate and 95% confidence interval (95% CI). If there is significant heterogeneity such as research subjects and interventions in the studies used to perform meta-analysis, these studies would not be directly combined. Statistical consistency was assessed using chi-square tests and I^2^ statistics for heterogeneity. If *p* > 0.1, no heterogeneity was considered. If *p* < 0.1, heterogeneity between studies was considered.

## Results

### Search and study selection

**Fig. 1:**
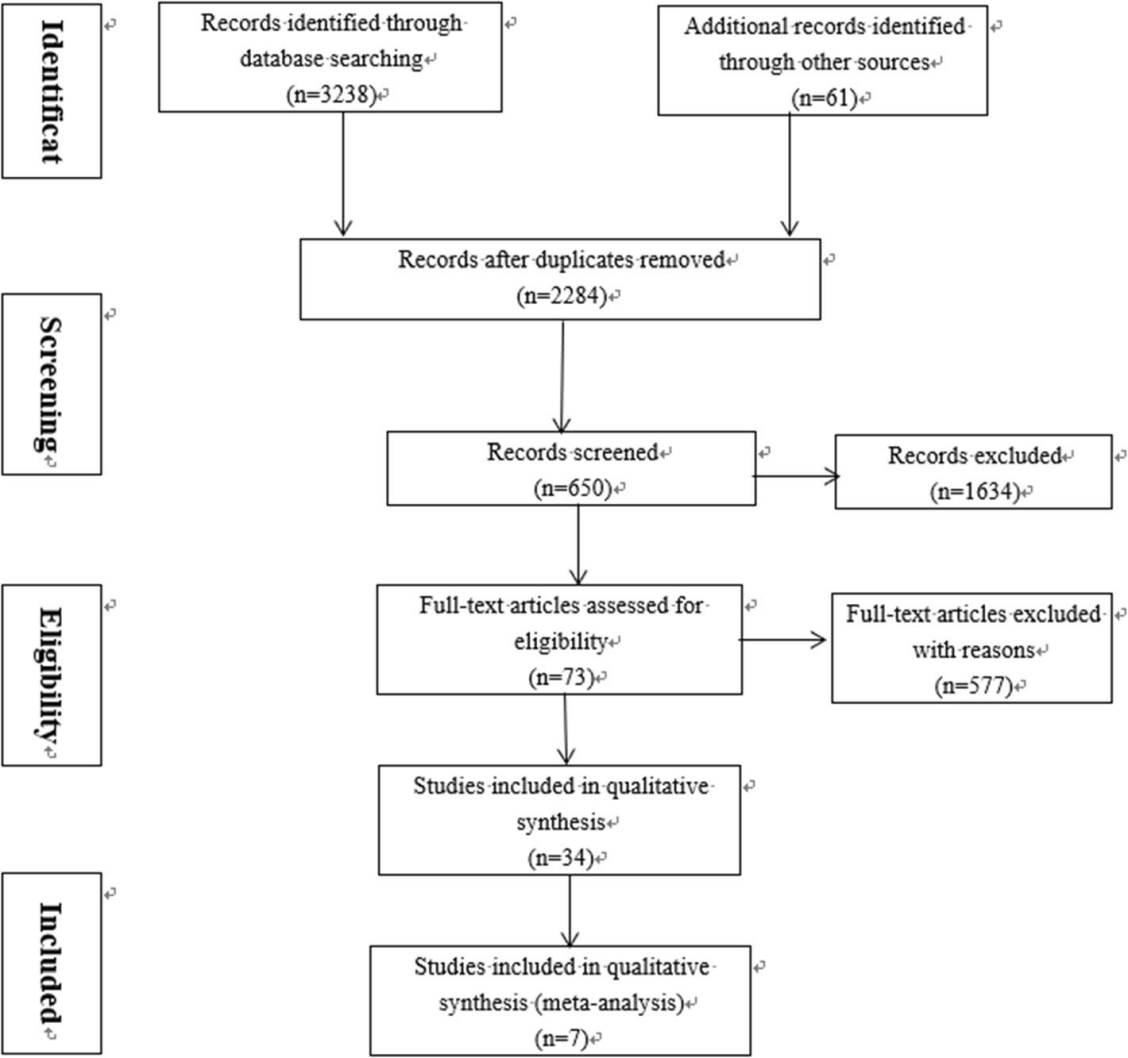
Selection of study

Three thousand two hundred thirty-eight documents were obtained through database searching with a manual search of 61 added references related to empirical researches on hospital pharmaceutical care. After removing duplicate articles, 2284 articles remained. Through reviewing titles and abstracts, 1634 irrelevant articles were excluded. After reading full texts, 577 articles inconsistent with this study were excluded. And 73 studies deemed suitable were assessed and excluded after screening (Fig. 1). Finally, 42 studies were included for the meta-analysis.

### Summary of included studies

Relevant studies were published mainly in Europe countries and America. There were 16 studies from the United States and 5 studies from the United Kingdom; 5 studies from China; 3 studies from Australia; 2 studies from Germany and Netherlands; other studies from Singapore, Iran, France, Jordan, etc. Diseases interfered included hypertension, diabetes, nephropathy, etc. Since 2010, researches on effectiveness of hospital pharmaceutical care have greatly increased, especially in 2014. And diseases concerned shifted from traditional diseases with high-incidence to epidemic, chronic diseases. For observing changes after receiving pharmaceutical care from clinical pharmacists, most samples were inpatients. In terms of interventions, most pharmacist interventions were diverse. Patient education programs, physician advice, disease state monitor and management were referred to in most researches provided. As for effects of hospital pharmaceutical care, among the 42 articles included, 36 studies had positive effects, 5 studies had mixed effects, and one study had no effect.

### Methodological quality of studies

Of the 42 studies included, 17 studies belonged to high quality studies with scores of 3–4, and the remaining 23 studies were low-quality studies (1–2 score). Among 42 studies, there were 20 randomized controlled trials, 11 non-randomized controlled trials, and 11 cohort studies. Seventeen studies reported loss of withdrawal and 20 studies reported sample baselines, taking into account the effects of randomization, blinding, and allocation concealment on selection bias, implementation bias, and measurement bias.

### Meta-analysis

#### Meta-analysis of hospital pharmaceutical care on SBP

**Fig. 2:**
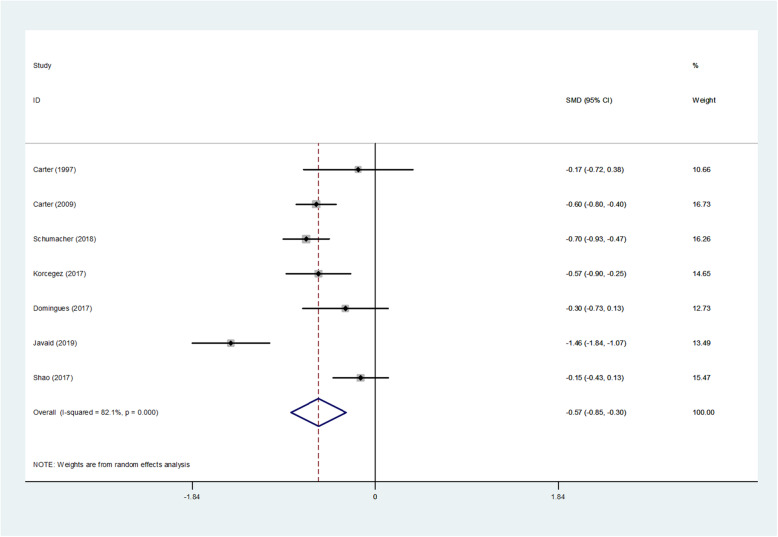
Forest figure of systolic blood pressure

A total of nine studies included blood pressure data, one of which missed standard deviation of the sample, and one experiment had an uneven baseline. Results of the meta-analysis of SBP by random effects model are shown in Table 2, Fig. 2. The results of SBP heterogeneity test were significant (I^2^ = 82.1%, *p* = 0.000 < 0.1). The test results showed *p* = 0.000 < 0.05, indicating that hospital pharmaceutical care had a significant effect on the reduction of SBP, compared to usual care. The mean difference of SBP between the intervention groups and control groups was − 0.573 (95% CI, − 0.851 to − 0.295).

**Table 2:** Results of Meta-analysis of systolic blood pressure

Study	| SMD	[95% Conf. Interval]		% Weight
Carter (1997) [8]	| -0.173	− 0.723	0.377	10.66
Carter (2009) [15]	| -0.596	−0.796	− 0.396	16.73
Schumacher (2018)	| -0.696	−0.927	−0.465	16.26
Korcegez (2017)	| -0.574	−0.899	− 0.250	14.65
Domingues (2017) [40]	| -0.301	−0.732	0.129	12.73
Javaid (2019) [42]	| -1.456	−1.844	−1.067	13.49
Shao (2017)	| -0.148	−0.426	0.131	15.47
D + L pooled RR	| -0.573	−0.851	−0.295	100.00

Heterogeneity chi-squared = 33.52 (d.f. = 6) *p* = 0.000.

I-squared (variation in SMD attributable to heterogeneity) =82.1%.

Estimate of between-study variance Tau-squared = 0.1096.

Test of SMD = 0: z = 4.04 *p* = 0.000.

The significance level of the combined effect quantity test: 0.05.

The significance level of the heterogeneity test: 0.1.

Heterogeneity test: *p* > 0.1, no heterogeneity was considered; *p* < 0.1, heterogeneity was considered.

#### Meta-analysis of hospital pharmaceutical care on DBP

**Fig. 3:**
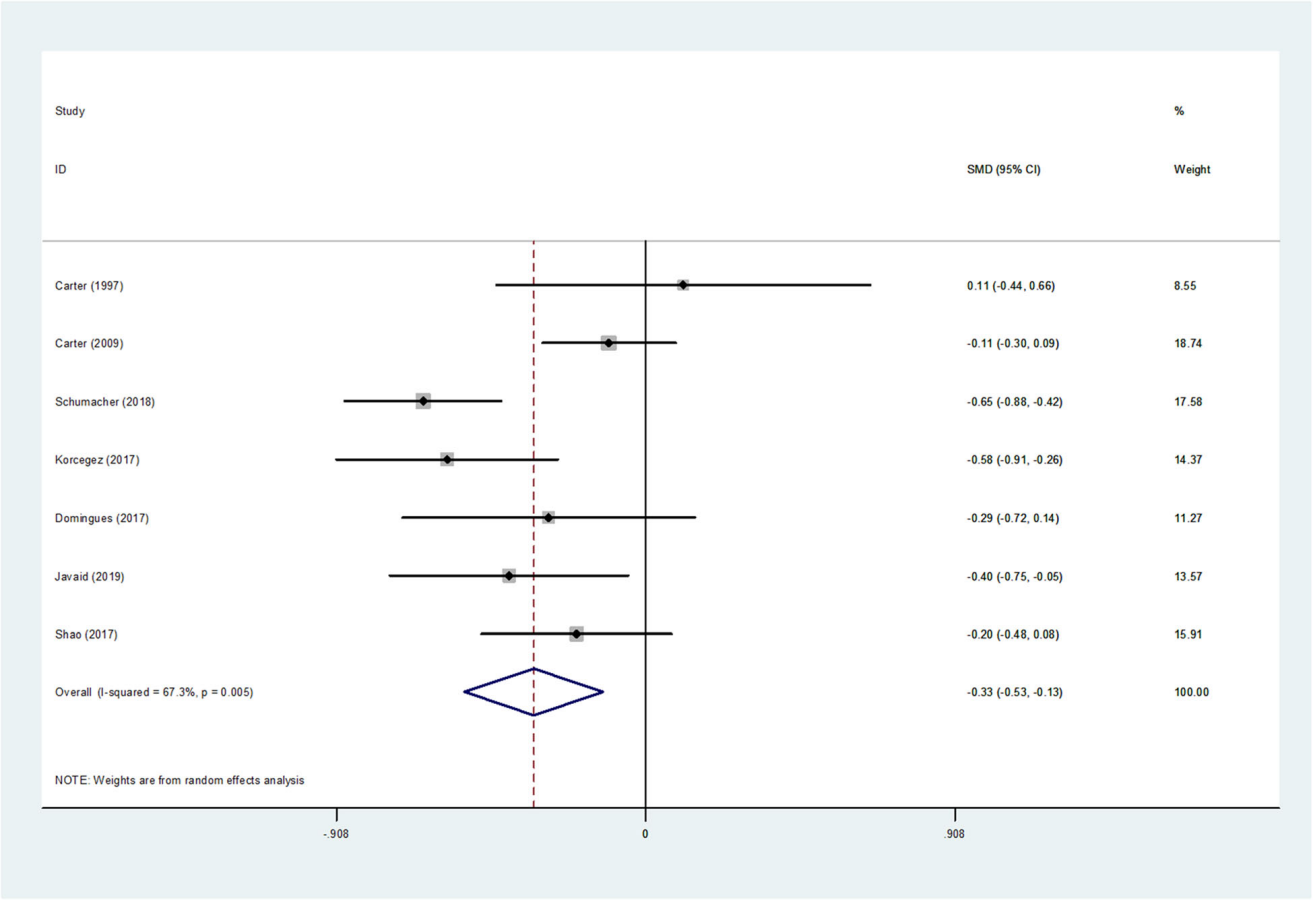
Forest figure of diastolic blood pressure

A total of nine studies included blood pressure data, one of which missed the standard deviation of the sample, and one experiment had an uneven baseline. Results of the meta-analysis of DBP by random effects model are shown as Table 3, Fig. 3. Heterogeneity test results on DBP were significant (I^2^ = 67.3%, *p* = 0.005 < 0.1). The test results showed that *p* = 0.002 < 0.05. It was shown that compared with usual care, hospital pharmaceutical care had significant effect on DBP. The average DBP difference between intervention group and control group was − 0.329 (95% CI, − 0.532 to − 0.125).

**Table 3:** Results of Meta-analysis of diastolic blood pressure

Study	| SMD	[95% Conf. Interval]		% Weight
Carter (1997) [8]	| 0.110	−0.439	0.660	8.55
Carter (2009) [15]	| -0.108	−0.304	0.087	18.74
Schumacher (2018)	| -0.654	−0.884	−0.424	17.58
Korcegez (2017)	| -0.584	−0.908	−0.259	14.37
Domingues (2017) [40]	| -0.285	−0.715	0.144	11.27
Javaid (2019) [42]	| -0.402	−0.752	−0.052	13.57
Shao (2017)	| -0.203	−0.482	0.075	15.91
D + L pooled SMD	| -0.329	−0.532	−0.125	100.00

Heterogeneity chi-squared =18.35 (d.f. = 6) *p* = 0.005.

I-squared (variation in SMD attributable to heterogeneity) = 67.3%.

Estimate of between-study variance Tau-squared = 0.0476.

Test of SMD = 0: z = 3.17 *p* = 0.002.

The significance level of the combined effect quantity test: 0.05.

The significance level of the heterogeneity test: 0.1.

Heterogeneity test: *p* > 0.1, no heterogeneity was considered; *p* < 0.1, heterogeneity was considered.

#### Meta-analysis of hospital pharmaceutical care on medical cost

**Fig. 4:**
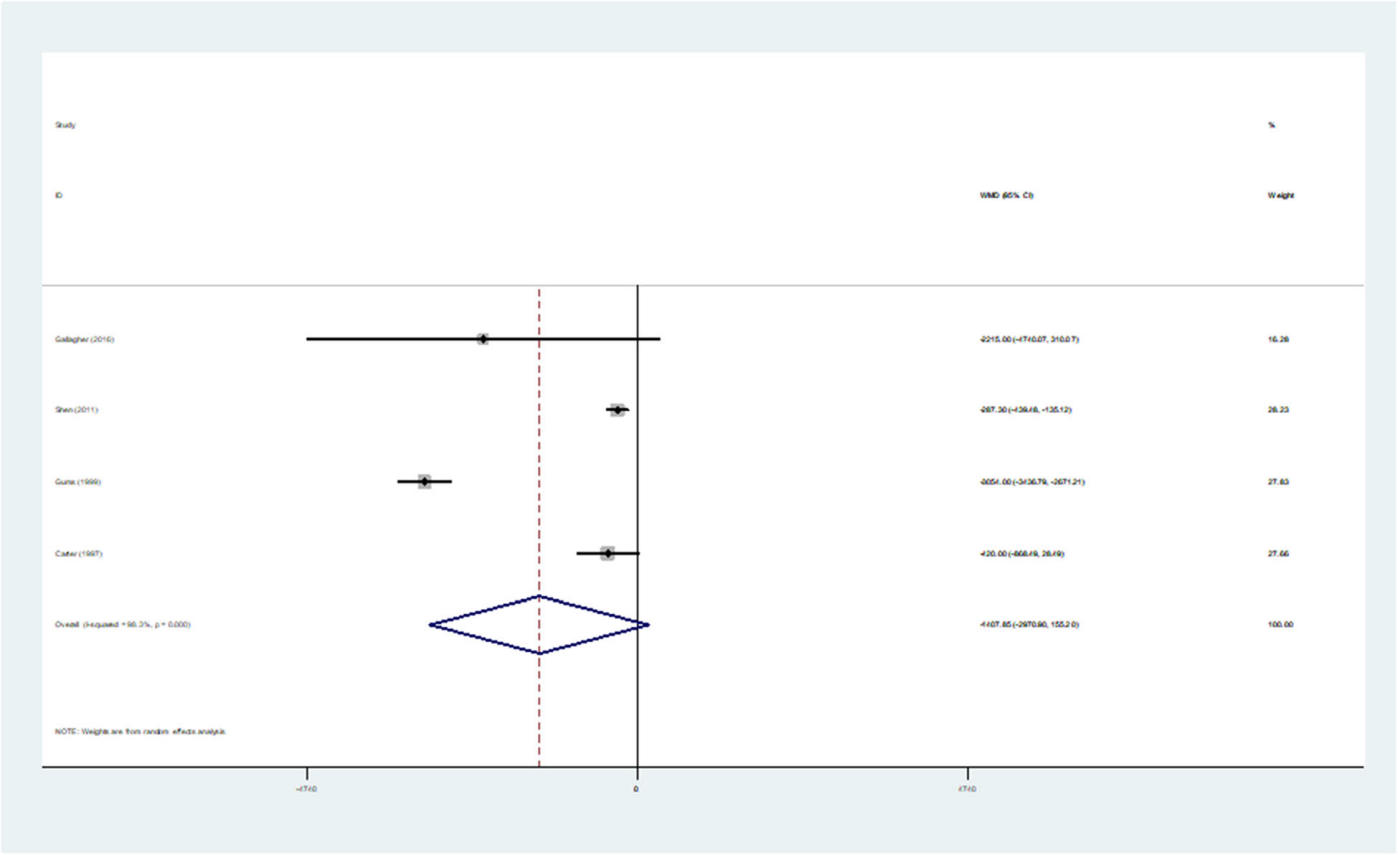
Forest figure of medical cost

A total of 15 studies included outcomes on patient medical costs, of which four experimental data missed sample standard deviations. Also, studies which had uneven baselines and did not report baselines were excluded. Here is the meta-analysis of the random effects model of medical cost indicators. The heterogeneity test of medical cost was significant (I^2^ = 98.3%, *p* = 0.000 < 0.1). The test results showed that *p* = 0.078 > 0.05, indicating that compared with usual care, hospital pharmaceutical care was not statistically significant on reducing medical cost. Therefore, it is not strong enough to support positive economic effect of this care on reducing the cost of patient care (Table 4, Fig. 4).

**Table 4:** Results of Meta-analysis of medical cost

Study	| SMD	[95% Conf. Interval]		% Weight
Gallagher (2016) [33]	|-2.2e+ 03	−4.7e+ 03	310.072	16.28
Shen (2011) [18]	|-287.300	−439.475	− 135.125	28.23
Gums (1999) [10]	| − 3.1e+ 03	-3.4e+ 03	−2.7e+ 03	27.83
Carter (1997) [8]	|-420.000	− 868.487	28.487	27.66
D + L pooled WMD	|-1.4e+ 03	−3.0e+ 03	155.203	100.00

Heterogeneity chi-squared = 175.82 (d.f. = 3) p = 0.000.

I-squared (variation in WMD attributable to heterogeneity) =98.3%.

Estimate of between-study variance Tau-squared = 2.2e+ 06.

Test of WMD = 0: z = 1.77 *p* = 0.078.

The significance level of the combined effect quantity test: 0.05.

The significance level of the heterogeneity test: 0.1.

Heterogeneity test: *p* > 0.1, no heterogeneity was considered; *p* < 0.1, heterogeneity was considered.

#### Meta-analysis of hospital pharmaceutical care on hospitalization days

**Fig. 5:**
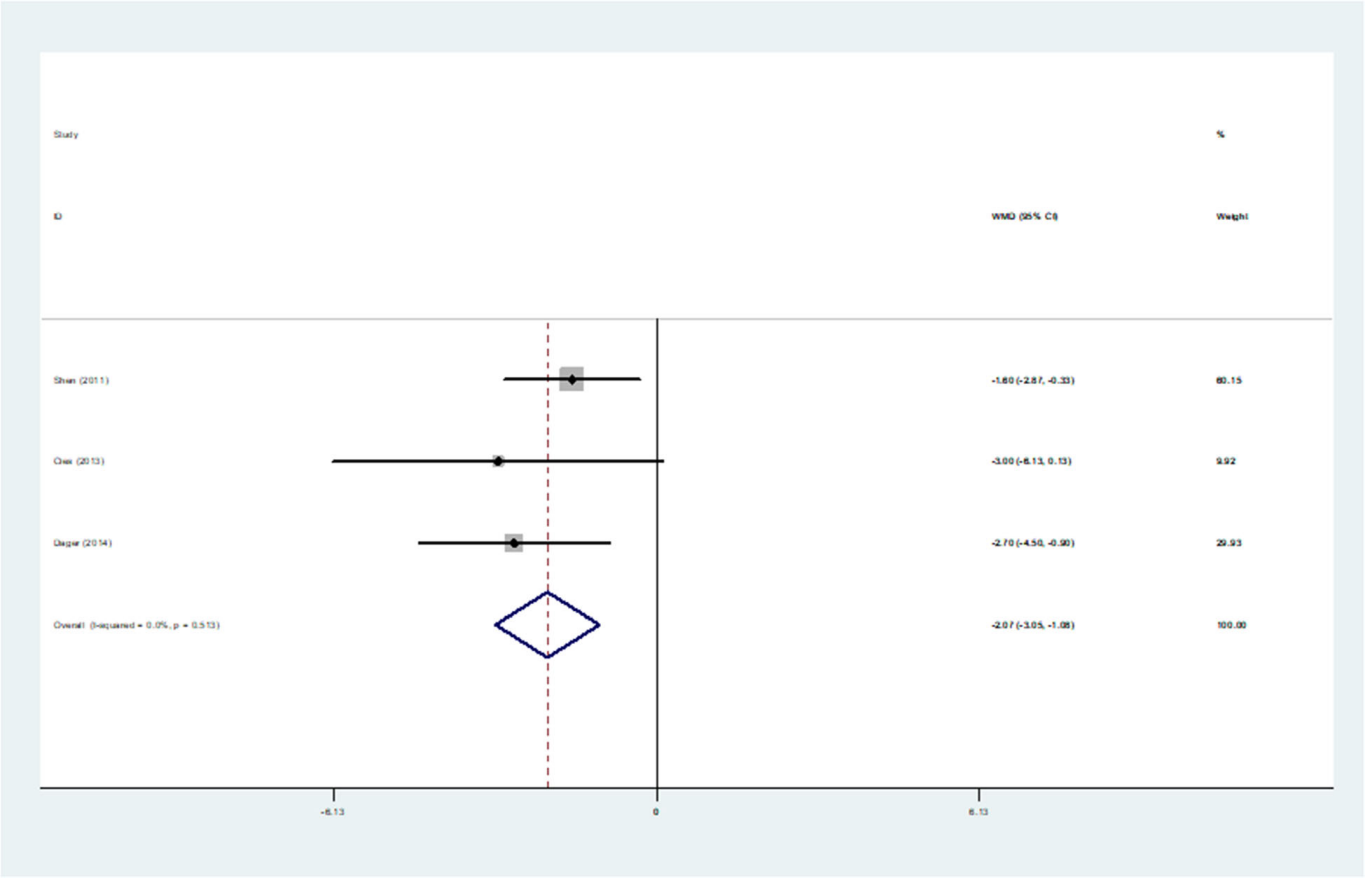
Forest figure of hospitalization days

A total of 11 studies covered patient days of hospitalization, of which four experimental data missed sample standard deviations and four experiments had an uneven baseline. The following is the result of a meta-analysis on the random effects model of hospital stay days. The heterogeneity test of the hospitalization days was significant (I^2^ = 0.0%, *p* = 0.513 > 0.1). The test results showed that *p* = 0.000 < 0.05, indicating that compared with usual care, hospital pharmaceutical care could reduce hospital stay significantly, and the average length of stay between intervention group and control group was − 2.068 (95% CI, − 3.054 to − 1.082) (Table 5, Fig. 5).

**Table 5:** Results of Meta-analysis of hospitalization days

Study	| SMD	[95% Conf. Interval]		% Weight
Shen (2011) [18]	| -1.600	−2.871	−0.329	60.15
Cies (2013) [21]	| -3.000	−6.131	0.131	9.92
Dager (2014)	| -2.700	−4.502	−0.898	29.93
I-V pooled WMD	| -2.068	−3.054	−1.082	100.00

Heterogeneity chi-squared = 1.33 (d.f. = 2) p = 0.513.

I-squared (variation in WMD attributable to heterogeneity) = 0.0%.

Test of WMD = 0: z = 4.11 *p* = 0.000.

The significance level of the combined effect quantity test: 0.05.

The significance level of the heterogeneity test: 0.1.

Heterogeneity test: *p* > 0.1, no heterogeneity was considered; *p* < 0.1, heterogeneity was considered.

## Discussion

This study systematically evaluated the clinical and economic outcomes of hospital pharmaceutical and conducted a meta-analysis. This study conducted a systematic review and meta-analysis of the clinical and economic outcomes of hospital pharmaceutical care. From Table 1, the vast majority of the studies showed that clinical pharmacy interventions could improve the economic and clinical outcomes, playing a significant role in improving medication errors, reducing readmission rates, and reducing medication costs. Among the 42 studies included, the primary outcomes of this service showed positive effects, among which 36 experimental groups were significantly better than their control groups, and the remaining 6 studies showed mixed or no effect.

Overall, hospital pharmaceutical care showed positive clinical outcomes. Results of the meta-analysis showed that the intervention of pharmaceutical care had a significant effect on the reduction of SBP and DBP. Meanwhile, results of the meta-analysis showed that hospital pharmaceutical care had a significant impact on hospitalization days, but no significant effect on reducing medical cost. In an academic medical intensive care unit, a randomized controlled trial was conducted on 202 patients before the intervention and 162 patients after the intervention. This study showed that the administration of medications by the pharmacist team effectively reduced inappropriate stress of ulcer prophylaxis use [20], finally leading to reduced medical cost (*p* = 0.000). It might be attributed to insufficient number of relevant studies, or different calculation methods and scope for medical cost in various studies. In Carter’s research, costs associated with prescriptions and visits as well as the total cost per patient were evaluated, but no specific cost items were listed. While in Gallagher’s study [34], medical expenses covered expenses of pharmacist, non-consultant hospital physicians, senior staff nurses, inpatient days, software costs and training costs. Although studies of Carter et al. [9] and Gum [13] reported positive economic effects, their sample sizes were not large enough to support its effectiveness. The small sample size was also one of the reasons for the lack of significant results.

This study has certain limitations. First, high-quality studies and total number of studies included for meta-analysis is insufficient. Researches on pharmaceutical care carried out in hospitals with strict study design are to be updated. Second, it is difficult to determine which intervention(s) of hospital pharmaceutical care caused specific effects. How much beneficial certain pharmacy services are than other pharmacy services might be the potential problem to be settled in the future.

## Conclusion

The results of meta-analysis showed that the hospital pharmaceutical care had a significant effect on reducing SBP, DBP and hospital stay, but no significant reduction on medical cost. In addition, because the data available for meta-analyses are not sufficient, a false-negative conclusion could be easily drawn. Therefore, hospital pharmaceutical care have a positive clinical and economic elimination in terms of reducing SBP, DBP and improving patient hospital stay, but follow-ups on medical cost as well as other outcomes need more experimental data to support.

## Data Availability

The datasets used and/or analysed during the current study are available from the corresponding author on reasonable request.

## Abbreviations

SBP: Systolic blood pressure
DBP: Diastolic blood pressure
SMD: Standard mean difference
WMD: Weighted mean difference

## References

[1] Draft statement on pharmaceutical care (1993). ASHP council on professional affairs. Am J Hosp Pharm.

[2] Wolf C, Pauly A, Mayr A (2015). Pharmacist-led medication reviews to identify an collaboratively resolve drug-related problems in psychiatry-a controlled, clinical trial. PLoS One.

[3] Zhang C, Zhang L, Huang L, Luo R, Wen J (2012). Clinical pharmacists on medical care of pediatric inpatients: a single-center randomized controlled trial. PLoS One.

[4] Xin C, Ge X, Yang X, Lin M, Jiang C, Xia Z (2014). The impact of pharmaceutical care on improving outcomes in patients with type 2 diabetes mellitus from China: a pre- and postintervention study. Int J Clin Pharm.

[5] Gallagher J, Mccarthy S, Byrne S (2014). Economic evaluations of clinical pharmacist interventions on hospital inpatients: a systematic review of recent literature. Int Jof Clin Pharm.

[6] Billi JE, Duranarenas L, Wise CG, Bernard AM, Mcquillan M, Stross JK (1992). The effects of a low-cost intervention program on hospital costs. J Gen Intern Med.

[7] Masters M, Krstenasky PM (1992). Positive effect of pharmaceutical care interventions in an internal medicine inpatient setting. Ann Pharmacother.

[8] Carter BL, Barnette DJ, Chrischilles E, Mazzotti GJ, Asali ZJ (1997). Evaluation of hypertensive patients after care provided by community pharmacists in a rural setting. Pharmacotherapy..

[9] Fraser GL, Stogsdill P, Dickens JD, Wennberg DE SR, Prato BS (1997). Antibiotic optimization. An evaluation of patient safety and economic outcomes. Arch Intern Med.

[10] Gums JG, Yancey RW (1999). HamiltonCA, Kubilis PS. A randomized, prospective study measuring outcomes after antibiotic therapy intervention by a multidisciplinary consult team. Pharmacotherapy..

[11] Dager WE, Branch JM, King JH (2000). Optimization of inpatient warfarin therapy: impact of daily consultation by a pharmacist-managed anticoagulation service. Ann Pharmacother.

[12] Canales PL, Dorson PG, Crismon ML (2001). Outcomes assessment of clinical pharmacy services in a psychiatric inpatient setting. Am J Health Syst Pharm.

[13] Brook O, Hout HV, Nieuwenhuyse H, Heerdink E (2003). Impact of coaching by community pharmacists on drug attitude of depressive primary care patients and acceptability to patients; a randomized controlled trial. Eur Neuropsychopharmacol.

[14] Bolas H, Brookes K, Scott M, McElnay J (2004). Evaluation of a hospital-based community liaison pharmacy service in Northern Ireland. Pharm World Sci.

[15] Carter BL, Ardery G, Dawson JD (2009). Physician and pharmacist collaboration to improve blood pressure control. Arch Intern Med.

[16] Wong YM, Quek YN, Tay JC (2011). Efficacy and safety of a pharmacist-managed inpatient anticoagulation service for warfarin initiation and titration. J Clin Pharm Ther.

[17] Hammad EA, Yasein N, Tahaineh L, Albsoul-Younes AM (2011). A randomized controlled trial to assess pharmacist- physician collaborative practice in the management of metabolic syndrome in a university medical clinic in Jordan. J Manag Care Pharm.

[18] Shen J, Sun Q, Zhou X (2011). Pharmacist interventions on antibiotic use in inpatients with respiratory tract infections in a chinese hospital. Int J Clin Pharm.

[19] Mousavi M, Dashtikhavidaki S, Khalili H, Farshchi A, Gatmiri M (2013). Impact of clinical pharmacy services on stress ulcer prophylaxis prescribing and related cost in patients with renal insufficiency. Int J Pharm Pract.

[20] Shah M, Norwood CA, Farias S, Ibrahim S, Chong PH, Fogelfeld L (2013). Diabetes transitional care from inpatient to outpatient setting: pharmacist discharge counseling. J Pharm Pract.

[21] Cies JJ, Varlotta L (2013). Clinical pharmacist impact on care, length of stay, and cost in pediatric cystic fibrosis (CF) patients. Pediatr Pulmonol.

[22] Ho CK, Mabasa VH, Leung VW, Malyuk DL, Perrott JL (2013). Assessment of clinical pharmacy interventions in the intensive care unit. Can J Hosp Pharm.

[23] Chilipko AA, Norwood DK (2014). Evaluating warfarin management by pharmacists in a community teaching hospital. Consult Pharm.

[24] Grimes TC, Deasy E, Allen A (2014). Collaborative pharmaceutical care in an irish hospital: uncontrolled before-after study. BMJ Qual Saf.

[25] Joost R, Dörje F, Schwitulla J, Eckardt KU, Hugo C (2014). Intensified pharmaceutical care is improving immunosuppressive medication adherence in kidney transplant recipients during the first post-transplant year: a quasi-experimental study. Nephrol Dial Transplant.

[26] Tan EC, Stewart K, Elliott RA, George J (2014). Pharmacist consultations in general practice clinics: the pharmacists in practice study. Res Soc Adm Pharm.

[27] Vervacke A, Lorent S, Motte S (2014). Improved venous thromboembolism prophylaxis by pharmacist-driven interventions in acutely ill medical patients in Belgium. Int J Clin Pharm.

[28] Zhang HX, Li X, Huo HQ, Liang P, Zhang JP, Ge WH (2014). Pharmacist interventions for prophylactic antibiotic use in urological inpatients undergoing clean or clean-contaminated operations in a chinese hospital. PLoS One.

[29] Campo M, Roberts GW, Cooter A (2015). Chronic obstructive pulmonary disease exacerbations, ‘sugar sugar’, what are we monitoring?. J Pharm Pract Res.

[30] Delpeuch A, Leveque D, Gourieux B, Herbrecht R (2015). Impact of clinical pharmacy services in a hematology/oncology inpatient setting. Anticancer Res.

[31] Obarcanin E, Nemitz V, Schwender H, Hasanbegovic S, Kalajdzisalihovic S (2015). Pharmaceutical care of adolescents with diabetes mellitus type 1: the Diadema study, a randomized controlled trial. Int J Clin Pharm.

[32] Burnett AE, Bowles H, Borrego ME, Montoya TN, Garcia DA, Mahan C (2016). Heparin-induced thrombocytopenia: reducing misdiagnosis via collaboration between an inpatient anticoagulation pharmacy service and hospital reference laboratory. J Thromb Thrombolysis.

[33] Gallagher J, O’Sullivan D, Mccarthy S (2016). Structured pharmacist review of medication in older hospitalised patients: a cost-effectiveness analysis. Drugs Aging.

[34] Khalil V, Declifford JM, Lam S, Subramaniam A (2016). Implementation and evaluation of a collaborative clinical pharmacist's medications reconciliation and charting service for admitted medical inpatients in a metropolitan hospital. J Clin Pharm Ther.

[35] Phatak A, Prusi R, Ward B (2016). Impact of pharmacist involvement in the transitional care of high-risk patients through medication reconciliation, medication education, and postdischarge call-backs (ipitch study). J HospMed.

[36] Waters CD, Myers KP, Bitton BJ, Torosyan A (2017). Reply: clinical pharmacist management of bacteremia in a community hospital emergency department. Ann Pharmacother.

[37] Sloeserwij VM, Hazen AC, Zwart DL, Leendertse AJ, Poldervaart JM, de Bont AA (2019). Effects of non-dispensing pharmacists integrated in general practice on medication-related hospitalisations. Br J Clin Pharmacol.

[38] Christie S, Golbarg M, Monique C (2018). The effect of clinical pharmacists on readmission rates of heart failure patients in the accountable care environment. J Manage Care Spec Pharm.

[39] Ilktac KE, Mesut S, Kutay D (2017). Effect of a pharmacist-led program on improving outcomes in patients with type 2 diabetes mellitus from northern Cyprus: a randomized controlled trial. J Manage Care Spec Pharm.

[40] Domingues EAM, Ferrit-Martín M, Calleja-Hernández, ángel M (2017). Impact of pharmaceutical care on cardiovascular risk among older HIV patients on antiretroviral therapy. Int J Clin Pharm.

[41] Andrea SO, Pedro A, Hincapié-García Jaime A (2017). Effectiveness of the Dader method for pharmaceutical care on patients with bipolar I disorder: results from the EMDADER-TAB study. J Manage Care Spec Pharm.

[42] Javaid Z, Imtiaz U, Khalid I, Saeed H, Khan RQ, Islam M (2019). A randomized control trial of primary care-based management of type 2 diabetes by a pharmacist in Pakistan. BMC Health Serv Res.

[43] Hua S, Guoming C, Chao Z (2017). Effect of pharmaceutical care on clinical outcomes of outpatients with type 2 diabetes mellitus. Patient Preference Adherence.

[44] Juanes A, Garin N, Mangues MA (2017). Impact of a pharmaceutical care programme for patients with chronic disease initiated at the emergency department on drug-related negative outcomes: a randomised controlled trial. Eur J Hosp Pharm.

